# Human neutrophils phagocytose and kill *Acinetobacter baumannii* and *A. pittii*

**DOI:** 10.1038/s41598-017-04870-8

**Published:** 2017-07-04

**Authors:** María Lázaro-Díez, Itziar Chapartegui-González, Santiago Redondo-Salvo, Chike Leigh, David Merino, David San Segundo, Adrián Fernández, Jesús Navas, José Manuel Icardo, Félix Acosta, Alain Ocampo-Sosa, Luis Martínez-Martínez, José Ramos-Vivas

**Affiliations:** 1 Instituto de Investigación Valdecilla IDIVAL, Santander, 39011 Spain; 2 Servicio de Microbiología, Hospital Universitario Marqués de Valdecilla, Santander, 39008 Spain; 3 New York University School of Medicine, New York, 10003 USA; 4 Servicio de Inmunología, Hospital Universitario Marqués de Valdecilla, Santander, 39008 Spain; 5 Departamento de Biología Molecular, Universidad de Cantabria, Santander, 39011 Spain; 6 Departamento de Anatomía y Biología Celular, Universidad de Cantabria, Santander, 39011 Spain; 7 Grupo de Investigación en Acuicultura, Universidad de Las Palmas de Gran Canaria, Gran Canaria, 35214 Spain; 8 Red Española de Investigación en Patología Infecciosa (REIPI), Instituto de Salud Carlos III, Madrid, 28029 Spain; 9 Unidad de Gestión Clínica de Microbiología, Hospital Universitario Reina Sofía, Córdoba, 14004 Spain; 10 Instituto Maimónides de Investigación Biomédica de Córdoba (IMIBIC), Córdoba, 14004 Spain

**Keywords:** Phagocytes, Bacterial host response, Cellular microbiology

## Abstract

*Acinetobacter baumannii* is a common cause of health care associated infections worldwide. *A. pittii* is an opportunistic pathogen also frequently isolated from *Acinetobacter* infections other than those from *A. baumannii*. Knowledge of *Acinetobacter* virulence factors and their role in pathogenesis is scarce. Also, there are no detailed published reports on the interactions between *A. pittii* and human phagocytic cells. Using confocal laser and scanning electron microscopy, immunofluorescence, and live-cell imaging, our study shows that immediately after bacteria-cell contact, neutrophils rapidly and continuously engulf and kill bacteria during at least 4 hours of infection *in vitro*. After 3 h of infection, neutrophils start to release neutrophil extracellular traps (NETs) against *Acinetobacter*. DNA in NETs colocalizes well with human histone H3 and with the specific neutrophil elastase. We have observed that human neutrophils use large filopodia as cellular tentacles to sense local environment but also to detect and retain bacteria during phagocytosis. Furthermore, co-cultivation of neutrophils with human differentiated macrophages before infections shows that human neutrophils, but not macrophages, are key immune cells to control *Acinetobacter*. Although macrophages were largely activated by both bacterial species, they lack the phagocytic activity demonstrated by neutrophils.

## Introduction

*Acinetobacter baumannii* has been extensively studied because infections caused by this pathogen have been associated with high morbidity and mortality rates^1, 2^. Also, their ability to survive in dry conditions and their resistance to disinfectants allows these microorganisms to survive in the hospital environment^3, 4^. Furthermore, this organism frequently presents multidrug or pan-resistance^5, 6^. Due to those three attributes (survival in the hospital environment, antimicrobial resistance and virulence) it is likely that this organism will gain even increasing importance in the near future. Among *Acinetobacter* genus, *A. pittii* is another clinically relevant species. The significant role of *A. pittii* in human infections and the emergence of resistant strains have also become a great medical concern^7–9^.

When *Acinetobacter* strains penetrate epithelial barriers and invade the host tissues, they first encounter the so-called “professional phagocytes”, macrophages and neutrophils. Professional phagocytes play a key role in host defence by engulfing and killing microorganisms. Little is known about the relative contribution of macrophages and neutrophils in the initial phase of encounter with *Acinetobacter* strains.

Neutrophils (also known as polymorphonuclears, PMNs) are the most abundant leukocytes in the blood which are rapidly recruited to the inflammatory site upon inflammation. Neutrophils can eliminate microbes using three basic strategies: phagocytosis, degranulation, and by a recently discovered mechanism called NETosis, a specific type of cell death different from both necrosis and apoptosis^10–13^. Bacterial metabolites and inflammatory stimuli induce NETosis and the release of neutrophil extracellular traps (NETs). NETs are released to the extracellular space by activated neutrophils, but additional studies are required to establish under what conditions NETs play an important role in bacterial killing. Importantly, some pathogens are able to overcome these bactericidal mechanisms^14–18^.

In this study, we investigated the interaction of *A. baumannii* and *A. pittii* clinical isolates with professional phagocytes. Understanding the mechanisms by which *Acinetobacter* interacts with immune cells is a prerequisite for the development of new prophylactic or therapeutic agents to treat the infections caused by these bacteria. Therefore, the aim of this work was to clarify the mechanisms of host-microbe interaction between neutrophils and *Acinetobacter* with focus on phagocytosis and neutrophil extracellular traps release.

## Results

### Phagocytosis and clearance of *Acinetobacter* strains by human neutrophils

**Figure 1:**
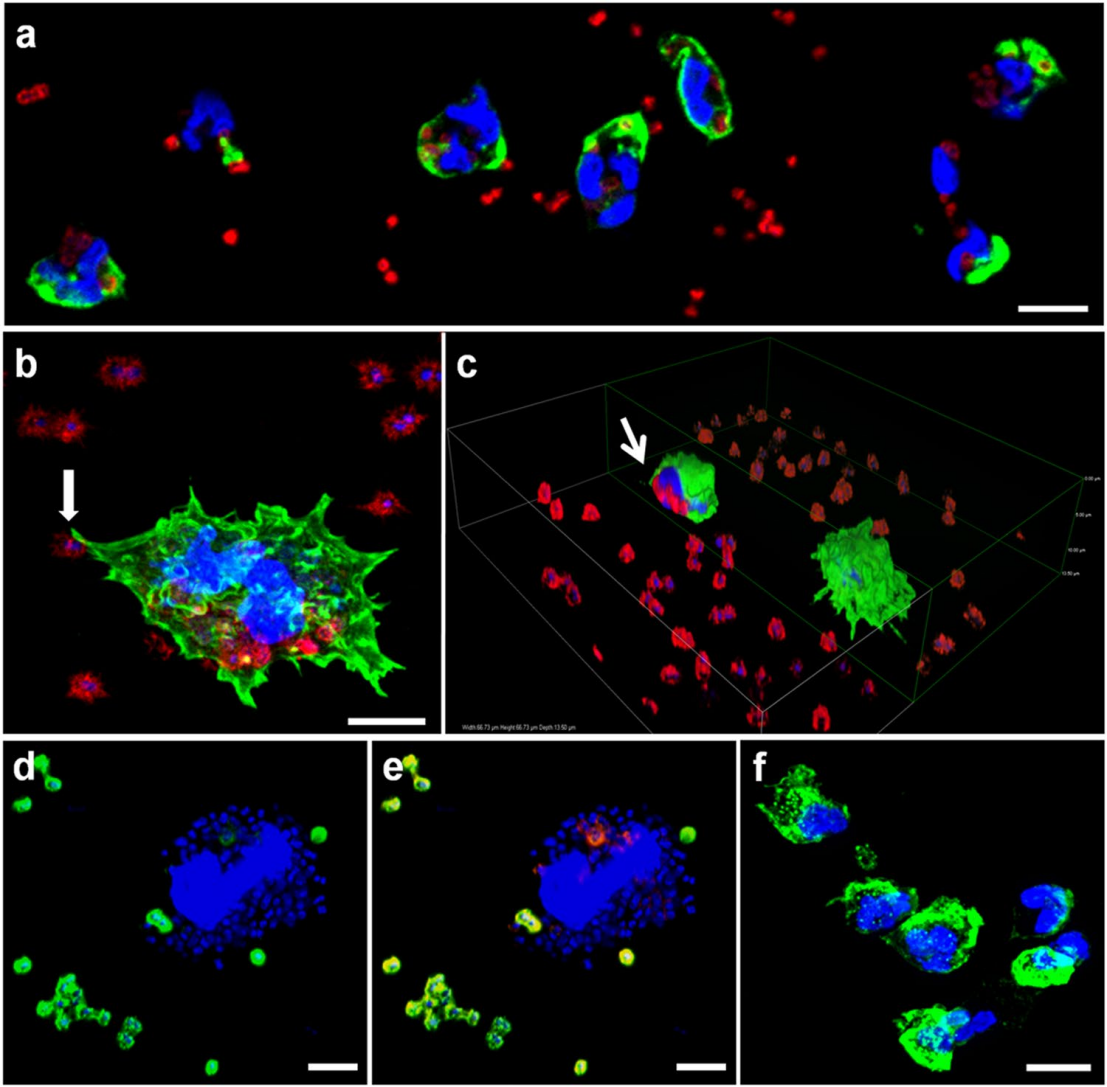
Contact and phagocytosis of *Acinetobacter* by human neutrophils. Human neutrophils were infected for 30 min (**a**), 60 min (**b,c**) or 2 h (**d–f**) with *A. baumannii* ATCC 19606^T^, fixed and processed for immunofluorescence labelling. Bacteria were detected with anti-*A. baumannii* rabbit antibody (red). Actin cytoskeleton was labelled with Atto 488 phalloidin (green) and nuclei are stained with DAPI (blue) (**a–c**,**f**). (**a**) Single stack; (**b** and **d–f**) maximal projections; (**c**) cross-sectional view. Arrow in (**b)** indicates a pseudopod in close contact with a bacterium. In (**d**,**e**) double-immunofluorescence images show extracellular bacteria (green), debris of intracellular bacteria (red) and bacterial and cellular DNA (blue). (**f**) As control, fresh untreated neutrophils were incubated in parallel during 4 h. Micrographs were originally captured at ×400 magnification (**a,f**) or ×600 magnification (**b–e**). Scale bars, (**a**,**f**) 5 µm; (**b,d,e**) 2 µm.

Human neutrophils are round cells that remain semi-attached and roll along the surfaces used in this study (glass or plastic). The presence of human (2%) or bovine serum (10%) in the protocol used to cultivate cells did not affect neutrophil behavior nor the outcome of the *in vitro* infections. The capability of neutrophils to bind and internalize *A. baumannii* and *A. pittii* is presented in Fig. 1. In presence of *Acinetobacter*, neutrophils can flatten and become phagocytic. The transition to active phagocytosis is sudden, with extension of the cell-bacteria contact area followed by the emergence of pseudopods to form a phagocytic arm that progresses to complete engulfment of the bacteria. Bacteria were associated with neutrophils as early as 30 min post-infection (Fig. 1a). Neutrophils were in contact with some of the surrounding bacteria through filopodia or pseudopods (arrow in Fig. 1b), and multiple attempts at phagocytosis were observed at neutrophil surfaces. At this time, whole bacteria (indicated by red fluorescence) were observed inside human neutrophils (Fig. 1c). After 2 h, bacteria already remained largely inside neutrophils, but with different sizes and some loss of their characteristic red immunofluorescent pattern, indicating that the phagocytosed bacteria were probably being degraded (Fig. 1d,e). Morphology in control neutrophils remains unchanged (Fig. 1f).

**Figure 2:**
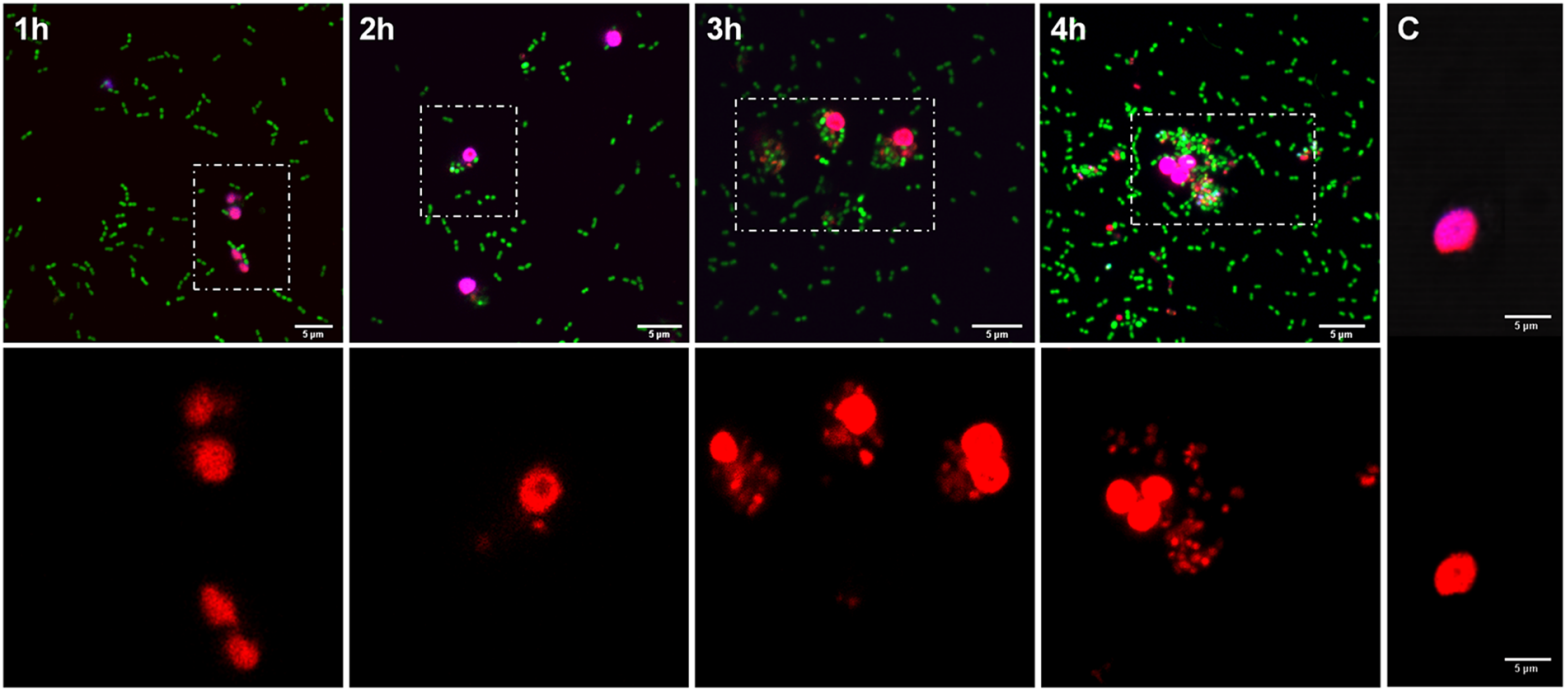
Live/Dead staining in unfixed neutrophils. Neutrophils were infected with *Acinetobacter* strains, then exposed to components of the live/dead kit, propidium iodide and SYTO9. Upper panels: in merged images, live bacteria appear green, dead bacteria appear red and eukaryotic nuclei appear pink. Lower panels show selected z-stacks at high magnifications (red channel) of the boxed areas in the upper panel. Untreated similarly stained cells served as control (C). Original magnifications: upper panels ×400; lower panels ×1000. Scale bars, 5 µm.

Furthermore, a Live/Dead staining was used to examine survival of *Acinetobacter* spp. after phagocytosis by unfixed primary human neutrophils. The dyes were added in the presence of 0.1% saponin, which sequesters cholesterol to preferentially permeabilize host cell plasma membranes, not *Acinetobacter* membranes. All acinetobacters stain with SYTO9, but only bacteria with compromised membranes stain with propidium iodide. The propidium iodide overcomes the SYTO9 fluorescence, so live bacteria appear green and dead bacteria appear red. Intracellular dead bacteria increased over time during the infection period (Fig. 2). From 2 to 4 h post-infection bacteria attached to plastic or glass surfaces divided rapidly and neutrophils tried to contain the bacterial overgrowth by quickly and continuously engulfing these pathogens (Supplementary videos 1 and 2).

**Figure 3:**
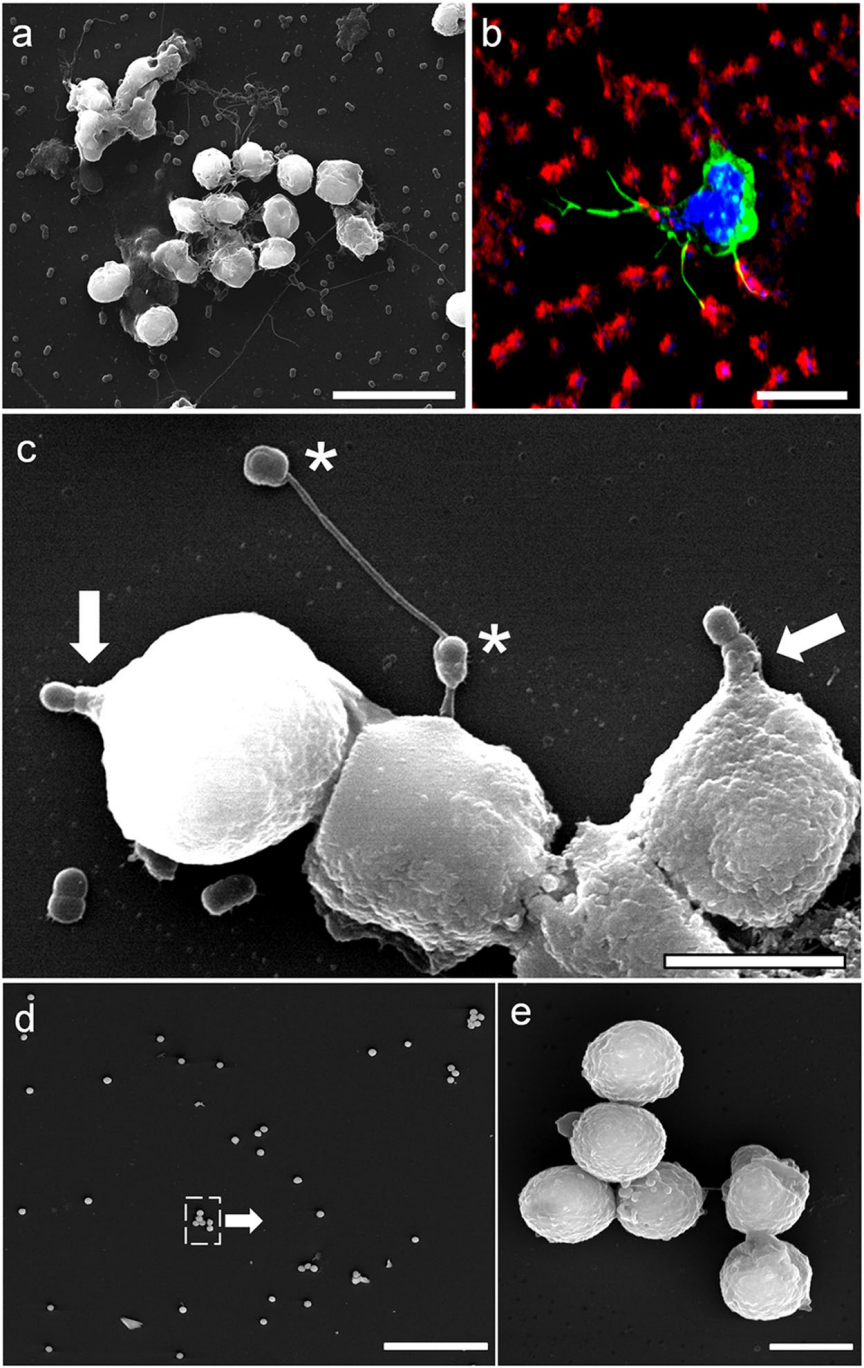
Capture and phagocytosis of *Acinetobacter* by human neutrophils. Pictures show SEM microphotographs (**a,c,d,e**) or immunofluorescence (**b**) images of infected neutrophils (3 h, strain ATCC 19606^T^). Large filopodia were observed in infected cultures in close contact with bacteria (**b,c**). Some of these filopodia completely surround two bacteria (asterisks in **c**) while pseudopods are catching bacteria attached to the inert surface (arrows in **c**). In (**b**) bacteria were detected with anti-*A. baumannii* rabbit antibody (red), actin cytoskeleton was labelled with Atto 488 phalloidin (green) and nuclei were stained with DAPI (blue). Unstimulated neutrophils show round shapes (**d**). (**e**) Detail of the boxed area in (**d**) Micrographs were originally captured at ×4000 (**a**), ×600 (**b**), ×10000 (**c**), ×500 (**d**) or ×9000 magnification (**e**). Scale bars, (**a**) 10 µm; (**b,c,e**) 5 µm; (**d**) 100 µm.

Interestingly, using scanning electron microscopy and immunofluorescence, we observed that human neutrophils used very large filopodia (more than 50 µm) to not only sense the environment, but also to detect and retain bacteria (Fig. 3). These large filopodia were also observed during experiments using live cell imaging on glass or plastic (Supplementary video 3).

Importantly, preincubation of neutrophils with actin-cytoskeleton inhibitor cytochalasin D abrogated phagocytosis of *Acinetobacter* strains. This was demonstrated by the presence of neutrophils without bacteria 3 h after infection (Supplementary Figure 1a,b). Of note, this cytoskeleton inhibitor reduces up to 90% of the number of neutrophils in the microscopic fields indicating that not only was phagocytosis affected, but also adherence of these cells to inert surfaces (the remained neutrophil morphology totally round).

Gentamicin protection assays also demonstrated that intracellular bacteria had died because no live bacteria were recovered 3 h after infections following gentamicin treatment (Supplementary Figure 1c). After performing quantitative CFUs counting experiments, difference in numbers between wells containing *Acinetobacter* and wells containing *Acinetobacter* plus neutrophils was not significative, despite neutrophils are able to eat at least 50 bacteria/cell (as observed by confocal microscopy) after 4 h of infection (Supplementary Figure 1d).

We incubated human neutrophils cells with extracellular products (ECPs) produced by all the *Acinetobacter* strains during growth in liquid medium, and no cytotoxicity was observed after 5 h of incubation with increasing volumes of bacterial ECPs (not shown).

### Production of neutrophil extracellular traps

**Figure 4:**
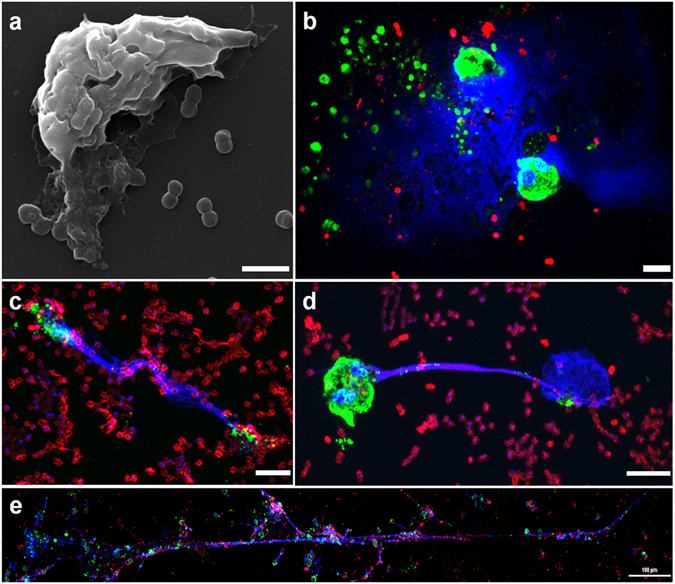
NETs production by human neutrophils infected with *Acinetobacter*. Pictures show a SEM microphotograph (**a**) or immunofluorescence images (**b–e**) of neutrophils 4 h post-infection: (**a**,**c–e**) strain ATCC 19606^T^; (**b**) strain HUMV 06-2790. From (**b** to **e**) bacteria were detected with anti-*A. baumannii* rabbit antibody (red), actin was labelled with Atto 488 phalloidin (green), and DNA was stained with DAPI (blue). Micrographs were originally captured at ×15000 (**a**), ×600 (**b,d**), ×400 (**c**) or ×200 magnification (**e**). Scale bars, (**a–d**) 5 µm; (**e**) 100 µm.

Neutrophils that had become engorged with microbes (some neutrophils were shown to harbour more than 50 bacteria) started to die after 3 h post infection (Fig. 4a,b). Neutrophils started to lose their individual nuclear lobules resulting in globular or horseshoe shape structures. During their final stage, nuclear and cytoplasmic integrity was lost, and most cells finally round up again and finally release NETs (Fig. 4c,d). Very occasionally, NETs form large aggregates (up to 1 mm in length) (Fig. 4e). In many cases, NETs clearly seems to entrap bacteria (Supplementary Figure 2a and Supplementary video 4). Immunofluorescence analyses confirmed the co-localization of histones (H3) and neutrophil elastase (NE) in extracellular traps released from human neutrophils (Supplementary Figure 2b,c). These NETs appear to be flexible, and to emerge from the cell from which they originated (Supplementary Figure 3a). The presence of NETs in infected cultures was highly variable. Non-infected neutrophils were used as controls for immunofluorescence staining in the nucleus (colocalization with histone H3) and cytoplasm (intracellular neutrophil elastase), and, as expected, *Pseudomonas aeruginosa* PAO1 infection used as positive control induced NET formation (Supplementary Figure 3b–d).

**Figure 5:**
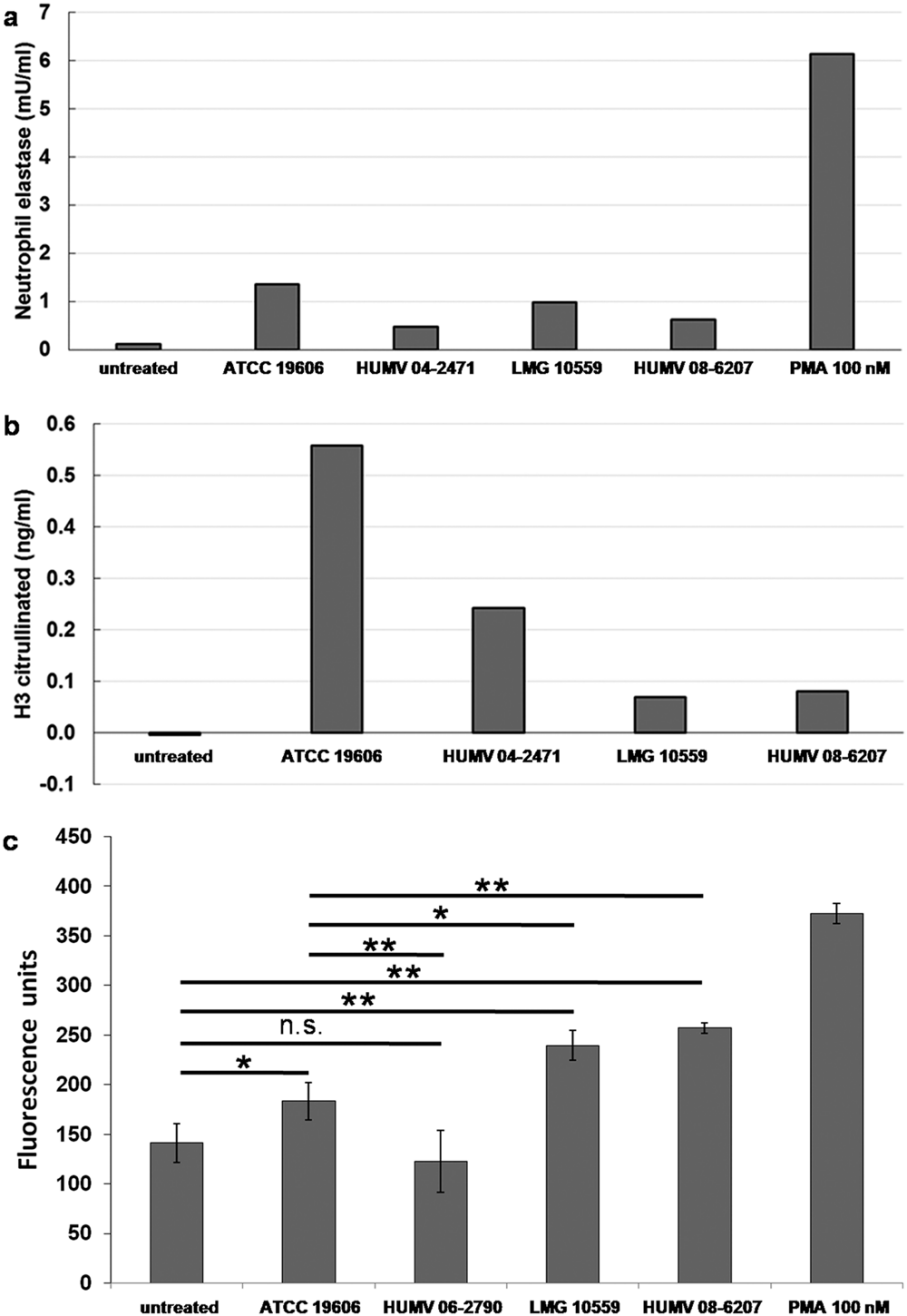
Quantification of elastase, citrullinated histone H3 and extracellular DNA using SYTOX Green. (**a**) Measurement of released neutrophil elastase. Human neutrophils were infected with *Acinetobacter* strains or treated with PMA for four hours, washed, and treated with S7 nuclease for 15 min. The supernatant from each well was assayed. Samples were tested in triplicate. (**b**) Measurement of citrullinated histone H3 (CitH3). Human neutrophils were infected with *Acinetobacter* strains for 4 hours. Supernatants were centrifuged to remove cellular debris and then tested in the ELISA. Samples were tested in triplicate. The concentrations of total neutrophil elastase and CitH3 in the analyzed samples were estimated from standard curves obtained for each assay. (**c**) Quantification of fluorescence after infection experiments using SYTOX Green. Supernatants from unstained infected cultures were partially digested with DNAse I and stained with SYTOX Green. Each bar indicates the average of three independent experiments ± SD. Asterisks indicate: *p = 0.0004; **p < 0.00001; n.s., p = 0.1610.

To quantify NETosis and NET release by *in vitro*-infected human neutrophils, neutrophil elastase and citrullinated histone H3 were measured by a NETosis assay and ELISA kit respectively. These assays demonstrated that *Acinetobacter* strains were able to induce the release of certain amounts of NETs by human neutrophils *in vitro* (Fig. 5a,b). However, NET release by infected neutrophils was always lower than neutrophils stimulated with the well known activator of full NETs release, PMA. To compare the induction of NETs by different strains, NETs formation was examined using the extracellular nucleic acid dye SYTOX Green by live-cell imaging during infections (Supplementary Figure 4). Furthermore, using a quantitative fluorescence assay, NETs formation by several strains was compared with untreated neutrophils and with neutrophils treated with PMA. Fluorescence from NETs in infected cultures with several *Acinetobacter* strains was also higher than in untreated neutrophils and lower that in PMA stimulated neutrophils. By this method, one strain (HUMV 06-2790) failed to clearly demonstrate NETs release (Fig. 5c).

### Infection of macrophage-neutrophil co-cultures

To test whether host cell type contributed mostly to clearance of this pathogen, we performed infections of mixed cultures containing human neutrophils and differentiated macrophages. Incubation of *Acinetobacter* with macrophages and neutrophils did not induce an important phagocytosis in macrophages, although produced remarkable important cell activation (compared with untreated macrophages), as demonstrated by the elongated cell shape. After 3 h of infection, >90% of macrophages were in contact with 5 or less bacteria despite that *Acinetobacter* was largely occupying the glass surface. On the other hand, neutrophils were full of bacteria (Supplementary Figure 5).

## Discussion

Neutrophils and macrophages are the first lines of defence against invading microbes. Neutrophils are terminally differentiated, rapidly reach the infection site, and are equipped with antimicrobial proteins to kill bacteria^19^. However, little is known about the relative contribution of neutrophils during the initial phase after encountering *Acinetobacter* spp. in human infections. Moreover, although several animal infection models were used to study the infection by *A. baumannii* (sepsis and lung infections), neutrophils from mammals and fish differ from human neutrophils in many ways^20–22^. As the success of *A. baumannii* and *A. pittii* as pathogens depends on its ability to avoid killing by components of the innate immune system, the aim of the current study was to characterize the human neutrophil response to these microbes. When neutrophils were assessed for their inherent abilities to neutralize *Acinetobacter* strains, both bacterial species were recognized within 20–30 min of co-incubation with cells. Immunofluorescence staining and double-immunofluorescence performed from 30 min to 4 h demonstrated that neutrophils catch bacteria continuously. This was also confirmed by time-lapse microscopy. Moreover, we examined live and dead acinetobacters inside neutrophils by using confocal microscopy. The primary goal of these experiments was to confirm whether the bacteria were located physically inside or outside the host cells and that dead bacteria inside cells lost their immunogenic surface (stained with a polyclonal antibody) because, although neutrophils kill the vast majority of bacteria, some microbes circumvent killing by these cells^14–16^. Using anti-*Acinetobacter* antibodies, whole bacteria were seen as red at the glass surface and associated with cells, but dead or damaged bacteria inside cells lost their characteristic red fluorescence. To unequivocally demonstrate that human neutrophils kill *Acinetobacter*, and therefore bacterial survival is compromised in presence of these cells, we performed an *in situ* Live/Dead staining on unfixed cells. This staining demonstrated that, once inside neutrophils, *Acinetobacter* die. This was observed along the experiments demonstrating that human neutrophils can easily and effectively kill both *A. baumannii* and *A. pittii in vitro*. In accordance with several authors, uptake of bacteria could lead to full activation of the anti-microbial arsenal of the neutrophil killing the ingested bacteria^23^. In conclusion, our findings strongly indicate that all the strains tested were phagocytosed and killed by human neutrophils. This is clearly in contrast to reports by others^24, 25^. Based on the experimental methods described in these previous publications, there is no obvious indication for the discrepancies in the reported results, apart from bacteria-cell contact time 1 h^26^ vs 4 h. According to our immunofluorescence, SEM, CFUs counting and live-cell and live/dead imaging experiments, neutrophils are in contact with *Acinetobacter* at 1 h, but further incubation time renders active phagocytosis. Our findings also correlate with current *in vivo* studies in mice and fish reporting the significance of neutrophils on *Acinetobacter* infections^26–28^. Moreover, our results correlate well with *in vitro* models using human neutrophils against other microbes, where phagocytosis seems to be the main mechanism to clear bacteria^10, 29^. Filopodia are abundant in macrophages^30^, but little is known about their role during phagocytosis or chemotaxis in neutrophils. An unexpected finding of the study was the presence of very large filopodia emerging from the neutrophil body to sense the environment and even to catch bacteria *in vitro*. Although quantitation of the filopodial dynamics or the cytoskeletal reorganization during neutrophil chemotaxis or phagocytosis is beyond the scope of this paper, new knowledge through a deeper study on the modulation and regulation of these filopodia may prove helpful in understanding the pathogenesis of this and other bacteria.

After 3–4 h post-infection, neutrophils started to die in presence of growing acinetobacters. In our assays, both *Acinetobacter* species grow actively in cell culture media and large numbers of bacteria were achieved 4 h after infections. Despite these *in vitro* assays did not allow new neutrophils recruitment, cells are full of dead bacteria 4 h after infections as demonstrate by confocal microscopy and gentamycin protection assays. This could mean that neutrophils play an important role against *Acinetobacter in vivo*.

Neutrophil cell death is fundamentally divided into necrosis, apoptosis, autophagy and the newly recognized NETosis. NETosis is a complex process that occurs with dramatic changes in the morphology of the neutrophil that finally lead to cell death^10^. The release of NETs against *Acinetobacter* was identical when human neutrophils were seeded on glass or plastic, as well as when using human or bovine serum. NETs are able to trap bacteria, fungi, and parasites^31^, but the possibility that the microbes ensnared in NETs are alive is controversial^32^. In our hands, *A. baumannii* and *A. pittii* induce a moderate cell death during the first 2 h of infection and NETs release by human neutrophils started after 3 h, similar to those induced by *P. aeruginosa*.

One of the most widely used techniques to observe NET induction is confocal microscopy. This approach is very informative as to the presence or absence of NETs, but microscopy images did not allow quantification of NETs. In this work, quantification of neutrophil elastase and citrullinated histone H3 demonstrated a strain-dependent variation in the NETs induction. Using SYTOX Green to stain and to quantify extracellular DNA, one strain failed to induce significant amounts of DNA release as compared with untreated controls. However, neutrophil extracellular traps release after *Acinetobacter* infections correlates with the presence of specific NETosis markers such as neutrophil elastase and histone H3^33^. Therefore, and in agreement with Naccache and Fernandes^33^ the experimental approaches to investigate NET formation underscore the need for consensus on standardized experimental approaches in the NET field.

Our results show that some bacteria were entrapped by NETs, and therefore this neutrophil response to these pathogens could partially prevent dissemination during the infection. A recent study shows that there are no *ex vivo* NETs production in neutrophils isolated from *Acinetobacter baumannii* bacteremia^34^. However, neither the presence of NETs *in vivo* was studied nor the neutrophil-*Acinetobacter* interactions *in vitro*.

Finally, using differentiated human macrophages in co-culture with neutrophils to study *Acinetobacter* host-microbe interactions, we show that neutrophils play a key role in controlling the infections caused by these bacteria. This is important because neutrophils make also an essential contribution in the recruitment and activation of macrophages during infections^35^. Our results also correlate with those of others showing that neutrophils, but not macrophages, are crucially to control early steps during bacterial and fungal infections^35, 36^.

In this work, our first objective was to demonstrate phagocytosis and killing of these two important pathogens by human neutrophils as a defence mechanism, but the induction of NETs in a small number of human neutrophils could be also important to fight infection. As neutrophils are also responsible for tissue damage and inflammation during certain circumstances, an overactivation of these cells (i.e. excessive NETs release) could be detrimental to the host. Therefore, future detailed studies at the molecular level will help to decipher the mechanisms involved in the regulation of neutrophils in presence of *Acinetobacter* or other pathogens, both alone or in combination with other immune cells.

## Methods

### Bacterial strains and growth conditions

The nine *Acinetobacter* clinical isolates (*A. baumannii* n = 4; *A. pittii* n = 5) used in this work were all previously described^37^. Reference strains *A. baumannii* ATCC 19606^T^ and *A. pittii* LMG 10559 were also included (Table 1). The strains were routinely cultured on blood agar (BA) plates, brain hearth infusion broth (BHIB) or Luria Bertani broth (LB) at 37 °C, and frozen at −80 °C with 20% glycerol. As control for NETs induction, *Pseudomonas aeruginosa* strain PAO1 was used^38^. *P. aeruginosa* was cultured in LB at 37 °C.

**Table 1:** *Acinetobacter* strains used in this study.

n°	Species	Strain	Clinical source
1	*A. baumannii*	^a^ATCC^®^ 19606^T^	urine
2	*A. baumannii*	^b^HUMV 1319	wound exudate
3	*A. baumannii*	HUMV 2471	sputum
4	*A. baumannii*	HUMV 2790	skin ulcer
5	*A. baumannii*	HUMV 3743	wound exudate
6	*A. pittii*	^c^LMG 10559	tracheal aspirate
7	*A. pittii*	HUMV 0315	sputum
8	*A. pittii*	HUMV 4336	diabetic foot exudate
9	*A. pittii*	HUMV 6207	wound exudate
10	*A. pittii*	HUMV 5918	wound exudate
11	*A. pittii*	HUMV 6483	urine

^a^ATCC, American Type Culture Collection. ^b^HUMV, Hospital Universitario Marqués de Valdecilla. ^c^LMG, Culture Collection of the Laboratorium voor Microbiologie Gent.

### Neutrophil isolation from whole human blood

All studies involving human samples were in accordance with international standards for research ethics and were approved by the local institutional review board (Hospital Universitario Marqués de Valdecilla). Neutrophils were isolated from whole venous blood obtained from healthy human volunteers after informed consent. The EasySep^TM^ Direct Human Neutrophil enrichment kit (StemCell) was used, following the manufacturer’s instructions. Briefly, 50 μL of EasySep^®^ neutrophil enrichment cocktail, containing a mix of tetrameric antibody complexes produced from monoclonal antibodies directed against the cell surface antigens CD2, CD3, CD9, CD19, CD36, CD56 and magnetic particles were added per 1 mL of blood. The blood/antibody/bead solution was adjusted to a total volume of 50 mL with recommended media and placed into an Easy 50 magnet for 10 min at room temperature (RT). Unbound neutrophils were pipetted into a new tube and placed in the Easy 50 magnet before addition of new magnetic particles. This step was repeated once. Highly-pure unbound neutrophils were briefly centrifuged and resuspended in RPMI 1640 media plus 10% fetal bovine serum (FBS) or 2% human serum. Neutrophils were also separated from other leukocytes using dextran density gradient centrifugation and red blood cells lysis as described elsewhere^39^. Neutrophils were isolated from samples from at least 14 donors and purity of neutrophil preparations was determined by morphology after staining of nuclei with NucBlue (Molecular Probes).

### Phagocytosis experiments

*Acinetobacter* strains were cultured overnight in 10 ml BHIB or LB at 37 °C with shaking at 175 rpm. Neutrophils were infected with bacteria at a multiplicity of infection (MOI, bacterium: eukaryotic cell ratio) of ~100:1. The number of colony forming units (CFUs) inoculated per well was determined by serial dilution in phosphate buffered saline (PBS) and plating on BA and incubated for 24 h. The infected plates were centrifuged for 4 min at 200 × *g* prior to the incubation to promote adherence of bacteria to cells and to synchronize infections. Infected cells were then incubated at 37 °C with 5% CO_2_ for different times. For quantification of live bacteria (extracellular and intracellular), external non-adherent bacteria were removed by washing four times with PBS, and human cells were then disrupted by addition of 100 µl Triton X-100 (1% in PBS) per well. To determine if *A. baumannii* is able to survive inside neutrophils after phagocytosis, strain *A. baumannii* ATCC 19606^T^ was selected. The MIC of gentamicin for this strain was previously determined^37^. Cells were infected for 2 h, washed with PBS, and the culture medium was replaced by medium containing 200 µg ml^−1^ of gentamicin (Gibco). Cells were incubated for a further 2 h, and lysed as described before. After this time, number of putative viable intracellular bacteria was counted. To do this, serial dilutions of the disrupted mixture were plated onto BA and incubated for 48 h at 37 °C. Growth of 3 *Acinetobacter* strains in presence or absence of neutrophils was monitored during 4 h. Viability/growth of *Acinetobacter* was calculated as the average of the total number of CFUs per total initial inoculum and expressed as a percentage. Quantitative phagocytosis experiments and growth experiments were repeated at least four times.

### Incubation with cytochalasin D

Neutrophils were incubated with the actin-cytoskeleton inhibitor cytochalasin D (5 µg ml^−1^) (Sigma) for 30 min before the bacteria were added. Neutrophils were then infected for 3 h as described for the immunofluorescence assays.

### Immunofluorescence assays

Cells were placed in 24-well tissue culture plates containing round glass coverslips. Bacteria were cultured as described above. Infected monolayers were incubated at 37 °C with 5% CO_2_ for different times (from 30 min up to 4 h). Cells were washed four times and fixed with cold paraformaldehyde (3.2% in PBS) for 20 min at room temperature. Then, cells were permeabilized with Triton X-100 (0.1% in PBS) for 5 min at RT and washed five times with PBS. Atto-488 phalloidin (Sigma), which binds polymerized F-actin, was used to identify actin filaments and fibers. Differential double immunofluorescent labelling of *Acinetobacter* allowed extracellular bacteria to be differentiated from intracellular bacteria. For double immunofluorescence assays, strains *A. baumannii* ATCC 19606^T^ and *A. pittii* LMG 10559 were used to produce polyclonal sera as previously described^40^. Antiserum was collected 8 weeks after the first boost, processed and stored using standard protocols^40^. Histones in NETs were stained with a rabbit polyclonal anti-histone H3 antibody (Abcam). Specific human neutrophil elastase was stained with an anti-neutrophil elastase rabbit monoclonal antibody (Abcam). Secondary antibodies conjugated to Alexa Fluor 594 or Alexa Fluor 488 goat anti-rabbit IgG were purchased from Invitrogen. After infections, coverslips were mounted on glass slides with Fluoroshield mounting medium containing DAPI (Sigma Aldrich) to stain double-stranded DNA. All preparations were examined with a Nikon A1R confocal scanning laser microscope equipped with 403 nm, 488 nm and 561 nm lasers. Images were captured at random with a ×20 Plan-Apo 0.75 NA, ×40 Plan-Fluor 1.3 NA or ×100 Apo-TIRF 1.49 NA objectives, and processed using the NIS-Elements 3.2 software. All immunofluorescence experiments for each strain were repeated with neutrophils from at least three different blood samples.

### Assessing Bacterial Viability inside neutrophils with Live/Dead staining

Bacterial viability inside neutrophils was determined by using the BacLight Live/Dead bacterial viability kit (Molecular Probes Inc.). Live/Dead Staining was performed in presence of 0.1% saponin for 20 min at 1 h, 2 h, 3 h and 4 h post-infection. A series of optical sections was obtained with a Nikon A1R confocal scanning laser microscope (CLSM); the excitation wavelengths were 488 nm (green) and 561 nm (red), and 500- to 550-nm and 570- to 620 nm emission filters were used, respectively. Images were captured at random with a 100× Apo TIRF (numerical aperture [NA], 1.49) objective. Reconstructions of confocal sections were assembled using NIS-Elements software, version 3.2.

### Time-lapse fluorescence microscopy

Time-lapse microscopy was carried out on a Nikon Eclipse Ti-E microscope (Nikon), equipped with a PlanFluor 20–40 × 0.6NA objective (Nikon) and a CO_2_ incubator. Neutrophils cells were seeded in 6-well plates (Nunc), in coated 4-well µ-slides (Ibidi, Martinsried, Germany) or in 24-well plates containing coverslips and infected as described before. NucBlue (one drop/well, Molecular Probes) or 10 µM SYTOX Green were added to each well to stain nuclei. Cells were infected as described before, and images were collected from 30 min up to 120 min post-infection every 2 min (NucBlue) or from 40 min up to 190 min post-infection every 1.5 min (SYTOX Green) with an ORCA- R2 CCD camera (Hamamatsu) powered by Nis Elements 3.2 software. For NucBlue, a 375–390 nm excitation, 420–490 nm emission filter was used and for SYTOX Green, a 485–520 nm excitation, 521/25 nm emission filter was used. Individual time-lapse frames were imported to the open source image analysis software, ImageJ (http://rsbweb.nih.gov/ij).

### NETosis assay

In separate experiments, we used a NETosis assay kit (Cayman Chemical) to determine the activity of NET-bound neutrophil elastase, according to manufacturer’s instructions. The assay is based on the enzymatic activity of neutrophil elastase in the culture medium that has been released from NETs through the action of S7 Nuclease. A colorimetric assay employing a specific elastase substrate (N-methoxysuccinyl-Ala-Ala-Pro-Val p-nitroanilide) was used after washing away non-NET associated elastase, as to measure only NET-associated elastase activity. The 5 substrate is selectively cleaved by elastase to give a 4-nitroaniline product that absorbs light at 405 nm. The concentration of neutrophil elastase was measured by optical densitometry in a Multiskan FC microplate reader (Thermo Fisher).

### Citrullinated Histone H3 assay

Quantitative determination of citrullinated histone was made using an ELISA Kit (citrullinated histone H3 ELISA kit, Cayman Chemical) according to manufacturer’s instructions. The concentration of citrullinated H3 was measured by optical densitometry at 450 nm in a Multiskan FC microplate reader (Thermo Fisher).

### Quantification of NET-DNA

Neutrophils were left untreated, treated with PMA (100 nM) or infected with *Acinetobacter* strains for 4 h. Wells containing infected cultures and controls were then treated with DNAse I (Sigma Aldrich) for 15 min at RT. The reaction was stopped with 0.5 M EDTA and cultures were centrifuged for 10 min at 8,000 × *g*. 150 µl supernatants from each well were transferred in triplicate into black 96-well plates (Thermo Scientific™). SYTOX Green was added (10 µM) to each well for 15 min and then fluorescence was quantified with excitation/emission wavelengths of 485/535 nm using a Synergy™ HTX Multi-Mode Microplate Reader (Biotek). All data were derived from three independent experiments. Statistical analysis of the data was carried out with the paired two-tailed Student t-test. A p-value less than 0.05 was considered statistically significant.

### Cytotoxicity of bacterial extracellular products

To determine the cytotoxic potential of the ECPs present in *Acinetobacter* culture supernatants, bacteria were grown on LB or BHIB for 24 h and collected by centrifugation at 3,000 rpm for 15 min at RT. The supernatants were sterilized via membrane filtration (0.22 µm, Millipore) and used immediately to challenge human neutrophils plated at density of 2 × 10^4^ cells/well. ECPs were added directly to the cell culture medium at different volumes (100–300 μl, each in duplicate) and cells were incubated for periods up to 24 h and processed for immunofluorescence. Control cultures were incubated with the same volumes using fresh bacterial culture medium.

### Scanning Electron Microscopy

Coverslips containing infected neutrophils were fixed in ice-cold 3% glutaraldehyde for 20 min at 4 °C. Samples were dehydrated with a graded ethanol series, dried by the critical point method, coated with gold in a Fine coat ion sputter JFC-1100 226 (JEOL, Ltd), and observed with an Inspect S microscope (FEI Company) working at 25 kV.

### Isolation and differentiation of macrophages from human blood

Human monocyte-derived macrophages (HMDM) were isolated from the peripheral blood of healthy donors as previously described. Briefly, blood was layered at a ratio of 2:1 (blood/Ficoll medium) on Ficoll Histopaque-1077 (Sigma) in 15 ml centrifuge tubes and spun for 30 min at 2000 rpm in an Allegra X-22R centrifuge (Beckman Coulter). The layer containing the peripheral blood mononuclear cells was collected and then resuspended in 15 ml of PBS, and recentrifuged for 10 min at 1000 rpm. After two washes in PBS, cells were resuspended in DMEM containing 10% FBS, L-Glutamine and 100 units ml^−1^ penicillin and 100 mg ml^−1^ streptomycin on 12 mm diameter coverslips in 24-well plates. Non-adherent cells were removed after 4 h. The cells were subsequently cultured in cell culture medium containing 50 ng ml^−1^ granulocyte macrophage colony stimulating factor (GM-CSF) (Sigma Aldrich) in an atmosphere containing 5% CO_2_. Cultures were fed daily, and infection experiments were performed 10 days after the peripheral blood was collected. Infections were performed with MOI of 100:1:1 (bacteria/neutrophil/macrophage) ratio.

## Electronic supplementary material

Supplementary Info

Supplementary video 1

Supplementary video 2

Supplementary video 3

Supplementary video 4

## References

[1] Clark NM, Zhanel GG, Lynch JP (2016). Emergence of antimicrobial resistance among *Acinetobacter* species: a global threat. Curr Opin Crit Care.

[2] Lahmer T (2014). *Acinetobacter baumannii* sepsis is fatal in medical intensive care unit patients: six cases and review of literature. Anaesth Intensive Care.

[3] Greene C, Vadlamudi G, Newton D, Foxman B, Xi C (2016). The influence of biofilm formation and multidrug resistance on environmental survival of clinical and environmental isolates of *Acinetobacter baumannii*. Am J Infect Control.

[4] Espinal P, Marti S, Vila J (2012). Effect of biofilm formation on the survival of *Acinetobacter baumannii* on dry surfaces. J Hosp Infect.

[5] Vila-Farres X (2012). *In vitro* activity of several antimicrobial peptides against colistin-susceptible and colistin-resistant *Acinetobacter baumannii*. Clin Microbiol Infect.

[6] Antunes LC, Imperi F, Minandri F, Visca P (2012). *In vitro* and *in vivo* antimicrobial activities of gallium nitrate against multidrug-resistant *Acinetobacter baumannii*. Antimicrobial agents and chemotherapy.

[7] Yamamoto M (2013). Regional dissemination of *Acinetobacter* species harbouring metallo-beta-lactamase genes in Japan. Clin Microbiol Infect.

[8] Pagano M (2015). Emergence of NDM-1-producing *Acinetobacter pittii* in Brazil. Int J Antimicrob Agents.

[9] Kamolvit W, Derrington P, Paterson DL, Sidjabat HE (2015). A case of IMP-4-, OXA-421-, OXA-96-, and CARB-2-producing *Acinetobacter pittii* sequence type 119 in Australia. J Clin Microbiol.

[10] Fuchs TA (2007). Novel cell death program leads to neutrophil extracellular traps. J Cell Biol.

[11] Brinkmann V, Zychlinsky A (2007). Beneficial suicide: why neutrophils die to make NETs. Nat Rev Microbiol.

[12] Standish AJ, Weiser JN (2009). Human neutrophils kill *Streptococcus pneumoniae* via serine proteases. J Immunol.

[13] Kumar V, Sharma A (2010). Neutrophils: Cinderella of innate immune system. Int Immunopharmacol.

[14] Greenlee-Wacker M, DeLeo FR, Nauseef WM (2015). How methicillin-resistant *Staphylococcus aureus* evade neutrophil killing. Curr Opin Hematol.

[15] Voyich JM (2005). Insights into mechanisms used by *Staphylococcus aureus* to avoid destruction by human neutrophils. J Immunol.

[16] Johnson MB, Criss AK (2011). Resistance of *Neisseria gonorrhoeae* to neutrophils. Front Microbiol.

[17] Kobayashi SD (2016). Phagocytosis and Killing of Carbapenem-Resistant ST258 *Klebsiella pneumoniae* by Human Neutrophils. J Infect Dis.

[18] Silva MT (2010). When two is better than one: macrophages and neutrophils work in concert in innate immunity as complementary and cooperative partners of a myeloid phagocyte system. J Leukoc Biol.

[19] Kaufmann SH, Dorhoi A (2016). Molecular Determinants in Phagocyte-Bacteria Interactions. Immunity.

[20] Rosen H (2013). Editorial: of mice and men–yet again. J Leukoc Biol.

[21] Mestas J, Hughes CC (2004). Of mice and not men: differences between mouse and human immunology. J Immunol.

[22] Bhuiyan MS (2016). *Acinetobacter baumannii* phenylacetic acid metabolism influences infection outcome through a direct effect on neutrophil chemotaxis. Proc Natl Acad Sci USA.

[23] Nordenfelt P, Tapper H (2011). Phagosome dynamics during phagocytosis by neutrophils. J Leukoc Biol.

[24] Kamoshida G (2015). *Acinetobacter baumannii* escape from neutrophil extracellular traps (NETs). J Infect Chemother.

[25] Kamoshida G (2016). A novel bacterial transport mechanism of *Acinetobacter baumannii* via activated human neutrophils through interleukin-8. J Leukoc Biol.

[26] Breslow JM (2011). Innate immune responses to systemic *Acinetobacter baumannii* infection in mice: neutrophils, but not interleukin-17, mediate host resistance. Infect Immun.

[27] van Faassen H (2007). Neutrophils play an important role in host resistance to respiratory infection with *Acinetobacter baumannii* in mice. Infect Immun.

[28] Guo B (2011). Quantitative impact of neutrophils on bacterial clearance in a murine pneumonia model. Antimicrobial agents and chemotherapy.

[29] Surewaard BG (2013). Staphylococcal alpha-phenol soluble modulins contribute to neutrophil lysis after phagocytosis. Cell Microbiol.

[30] Kress H (2007). Filopodia act as phagocytic tentacles and pull with discrete steps and a load-dependent velocity. Proc Natl Acad Sci USA.

[31] Lu T, Kobayashi SD, Quinn MT, Deleo FRA (2012). NET Outcome. Front Immunol.

[32] Menegazzi R, Decleva E, Dri P (2016). Killing by neutrophil extracellular traps: fact or folklore?. Blood.

[33] Naccache PH, Fernandes MJ (2016). Challenges in the characterization of neutrophil extracellular traps: The truth is in the details. European journal of immunology.

[34] Konstantinidis T (2015). Immunomodulatory Role of Clarithromycin in *Acinetobacter baumannii* Infection via Formation of Neutrophil Extracellular Traps. Antimicrobial agents and chemotherapy.

[35] Chertov O (1997). Identification of human neutrophil-derived cathepsin G and azurocidin/CAP37 as chemoattractants for mononuclear cells and neutrophils. J Exp Med.

[36] Mircescu MM, Lipuma L, van Rooijen N, Pamer EG, Hohl TM (2009). Essential role for neutrophils but not alveolar macrophages at early time points following *Aspergillus fumigatus* infection. J Infect Dis.

[37] Lazaro-Diez M (2016). *Acinetobacter baumannii* and *A. pittii* clinical isolates lack adherence and cytotoxicity to lung epithelial cells *in vitro*. Microbes Infect.

[38] Ocampo-Sosa AA (2012). Alterations of OprD in carbapenem-intermediate and -susceptible strains of *Pseudomonas aeruginosa* isolated from patients with bacteremia in a Spanish multicenter study. Antimicrobial agents and chemotherapy.

[39] Kuhns DB, Long Priel DA, Chu J, Zarember KA (2015). Isolation and Functional Analysis of Human Neutrophils. Curr Protoc Immunol.

[40] Ramos-Vivas J (2011). *Rhodococcus equi* human clinical isolates enter and survive within human alveolar epithelial cells. Microbes Infect.

