# Author Correction: Human neutrophils phagocytose and kill *Acinetobacter baumannii* and *A. pittii*

**DOI:** 10.1038/s41598-020-61184-y

**Published:** 2020-03-11

**Authors:** María Lázaro-Díez, Itziar Chapartegui-González, Santiago Redondo-Salvo, Chike Leigh, David Merino, David San Segundo, Adrián Fernández, Jesús Navas, José Manuel Icardo, Félix Acosta, Alain Ocampo-Sosa, Luis Martínez-Martínez, José Ramos-Vivas

**Affiliations:** 1 Instituto de Investigación Valdecilla IDIVAL, Santander, 39011 Spain; 2 Servicio de Microbiología, Hospital Universitario Marqués de Valdecilla, Santander, 39008 Spain; 3 New York University School of Medicine, New York, 10003 USA; 4 Servicio de Inmunología, Hospital Universitario Marqués de Valdecilla, Santander, 39008 Spain; 5 Departamento de Biología Molecular, Universidad de Cantabria, Santander, 39011 Spain; 6 Departamento de Anatomía y Biología Celular, Universidad de Cantabria, Santander, 39011 Spain; 7 Grupo de Investigación en Acuicultura, Universidad de Las Palmas de Gran Canaria, Gran Canaria, 35214 Spain; 8 Red Española de Investigación en Patología Infecciosa (REIPI), Instituto de Salud Carlos III, Madrid, 28029 Spain; 9 Unidad de Gestión Clínica de Microbiología, Hospital Universitario Reina Sofía, Córdoba, 14004 Spain; 10 Instituto Maimónides de Investigación Biomédica de Córdoba (IMIBIC), Córdoba, 14004 Spain

Correction to: *Scientific Reports* 10.1038/s41598-017-04870-8, published online 04 July 2017

Adrián Fernández was omitted from the author list in the original version of this Article. This has been corrected in the PDF and HTML versions of the Article, and in the accompanying Supplementary Information file.

The updated Author List now reads:

María Lázaro-Díez, Itziar Chapartegui-González, Santiago Redondo-Salvo, Chike Leigh, David Merino, David San Segundo, Adrián Fernández, Jesús Navas, José Manuel Icardo, Félix Acosta, Alain Ocampo-Sosa, Luis Martínez-Martínez & José Ramos-Vivas

The Author Contributions section now reads:

J.R.V. conceived the experiments, J.R.V and D.S.S. designed the experiments, M.L.D., I.C.G., S.R.S., C.L., D.M., A. F., F.A., A.O.S., J.M.I. and J.R.V. performed the experiments, M.L.D., I.C.G., J.N., F.A., J.M.I. L.M.M. and J.R.V. analyzed the data, A.O.S., D.S.S., J.N., F.A., J.M.I. contributed with reagents/materials/analysis tools, J.R.V. wrote the paper. All authors reviewed the manuscript.

