# Supplementary material for: Human neutrophils phagocytose and kill Acinetobacter baumannii and A. pittii
Source: Sci Rep. 2017 Jul 4;7:4571. doi: 10.1038/s41598-017-04870-8 (PMC5496873; doi:10.1038/s41598-017-04870-8)

Human neutrophils phagocytose and kill *Acinetobacter baumannii* and *A. pittii*

María Lázaro-Díez, Itziar Chapartegui-González, Santiago Redondo-Salvo, Chike Leigh, David Merino, David San Segundo, Adrián Fernández, Jesús Navas, José Manuel Icardo, Félix Acosta, Alain Ocampo-Sosa, Luis Martínez-Martínez and José Ramos-Vivas

Supplementary Figure 1. Cytochalasin and gentamicin treatments

Effects of pretreatment of human neutrophils with cytochalasin D (**a,b**). Neutrophils were infected with *A. baumannii* strain ATCC 19606^{T} for 3 h. Bacteria were detected with anti-*A. baumannii* rabbit antibody (red) and nuclei were stained with DAPI (blue). In merged images, actin cytoskeleton was detected with Atto 488 phalloidin (green). Micrograph was originally captured at ×400 magnification. Scale bars, 5 µm. **c**) Effects of the addition of gentamicin on bacterial survival in presence of neutrophils. Two hours after infections (MOI of 100:1), gentamicin was added. After 2 h post-treatment, the exact number of bacterial CFUs (as a percentage of the initial inoculum) was determined. Values represent means ± standard deviations from three independent experiments. G: gentamicin. **d**) Growth of *Acinetobacter* strains in presence or absence of neutrophils was monitored during 4 h. Viability/growth of Acinetobacter was calculated as the average of the total number of CFUs per total initial inoculum and expressed as a percentage. Black bars, *Acinetobacter* plus neutrophils; grey bars, *Acinetobacter* alone. Values represent means ± standard deviations from three independent experiments.

Supplementary Figure 2. **Traps colocalization with histone H3 and elastase.**

a) NETs (blue) colocalize with *Acinetobacter pittii* strain LMG-10559 (red). Immunofluorescence analyses confirmed the colocalization of histones (H3) (**b**) and neutrophil elastase (NE) (**c**) with DNA in extracellular traps released from human neutrophils. **b,** (from left to right) neutrophil elastase (green channel), DAPI (blue channel), and merged images. **c,** (from left to right) histone H3 (green), DAPI (blue) and merged images. Original magnification, **a**, ×600; **b**, **c** ×400. Scale bars: 5 µm.

Supplementary Figure 3.

NETs emerge from the cell from which they originated. Human neutrophils infected for 4 h with *A. pittii* strain HUMV 08-0315 (**a**). Bacteria were detected with anti-*A. baumannii* rabbit antibody (red), DNA was stained with DAPI (blue) and actin cytoskeleton was detected with Atto 488 phalloidin (green). (**b, c**) Control for antibody specificity in untreated neutrophils: anti-histone H3 (green) and nucleus (blue) (**b**); anti-neutrophil elastase (red), actin (green) and nucleus (blue) (**c**). NETs induced by *Pseudomonas aeruginosa* PAO1 (**d**), where bacteria were detected with anti-*P. aeruginosa* antibody (red), DNA was stained with DAPI (blue) and actin cytoskeleton was detected with Atto 488 phalloidin (green). Original magnifications, **a**, ×400; **b,c** ×600; **d**, ×400. Scale bars: **a,c**, 10 µm; **b,d**, 5 µm.

Supplementary Figure 4.

Live-cell experiments were performed in presence of SYTOX Green. Screenshots were taken from 40 min post- infection (time 0h) up to 190 min post-infection (time 4h). From left to right, untreated neutrophils, neutrophils infected with *Acinetobacter* and neutrophils treated with PMA.

Supplementary Figure 5. Infection of co-cultures of human neutrophils and macrophages. Co-cultures were infected for 3 h with *A. baumannii* strain ATCC 19606^T (**a-a´**) or *A. pittii* LMG 10559 (**b-b´**). After infections, cells were fixed and processed for immunofluorescence labeling and confocal microscopy. The image shows maximal projections where bacteria were detected with anti-*Acinetobacter* rabbit antibodies (red), actin cytoskeleton was labeled with Atto 488 phalloidin (green) and nuclei were stained with DAPI (blue). Arrows indicate macrophages and asterisks indicate neutrophils (**a,b**) or their location (**a´,b´**). Untreated differentiated human macrophages were included for shape comparison (C). Micrographs were originally captured at ×400 magnification. Scale bars, a-b´, 10 µm; C, 20 µm.

Supplementary videos 1 and 2.

Time-lapse microscopy showing active phagocytosis of *Acinetobacter* by human neutrophils. NucBlue (DNA) *ex vivo* staining was applied to show the multi-lobulated neutrophil nuclei. The video was recorded between 2 h and 3 h post infection.

Supplementary video 3.

Time-lapse microscopy of untreated neutrophils showing large filopodia.

Supplementary video 4.

Human neutrophils infected for 4 h with *A. baumannii* strain HUMV 07-1319. NETs emerge from the cell from which they originated to entrap bacteria. Bacteria were detected with anti-*A. baumannii* rabbit antibody (red), DNA was stained with DAPI (blue) and actin cytoskeleton was detected with Atto 488 phalloidin (green).

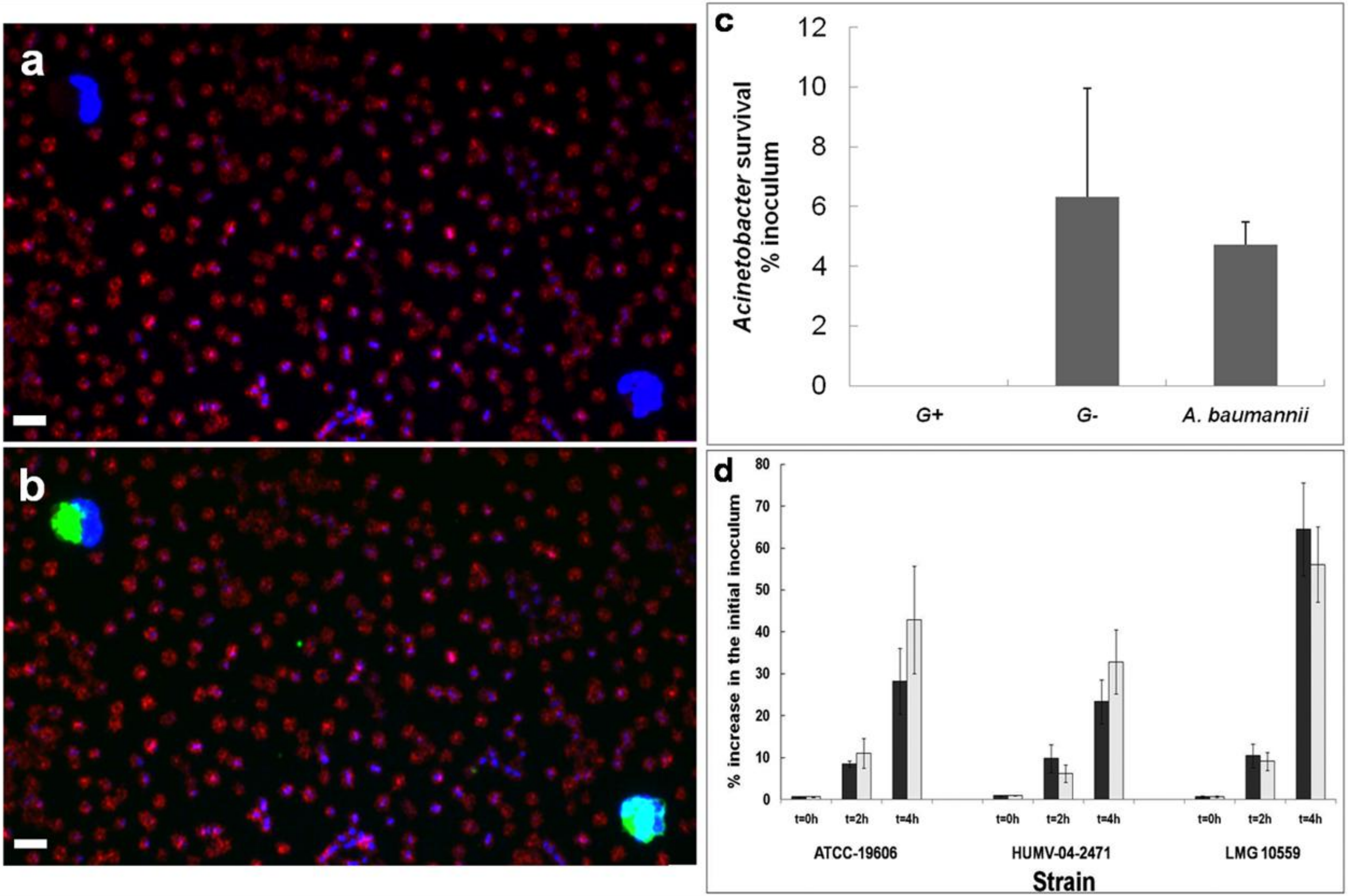

Supplementary Figure 1

Supplementary Figure 2

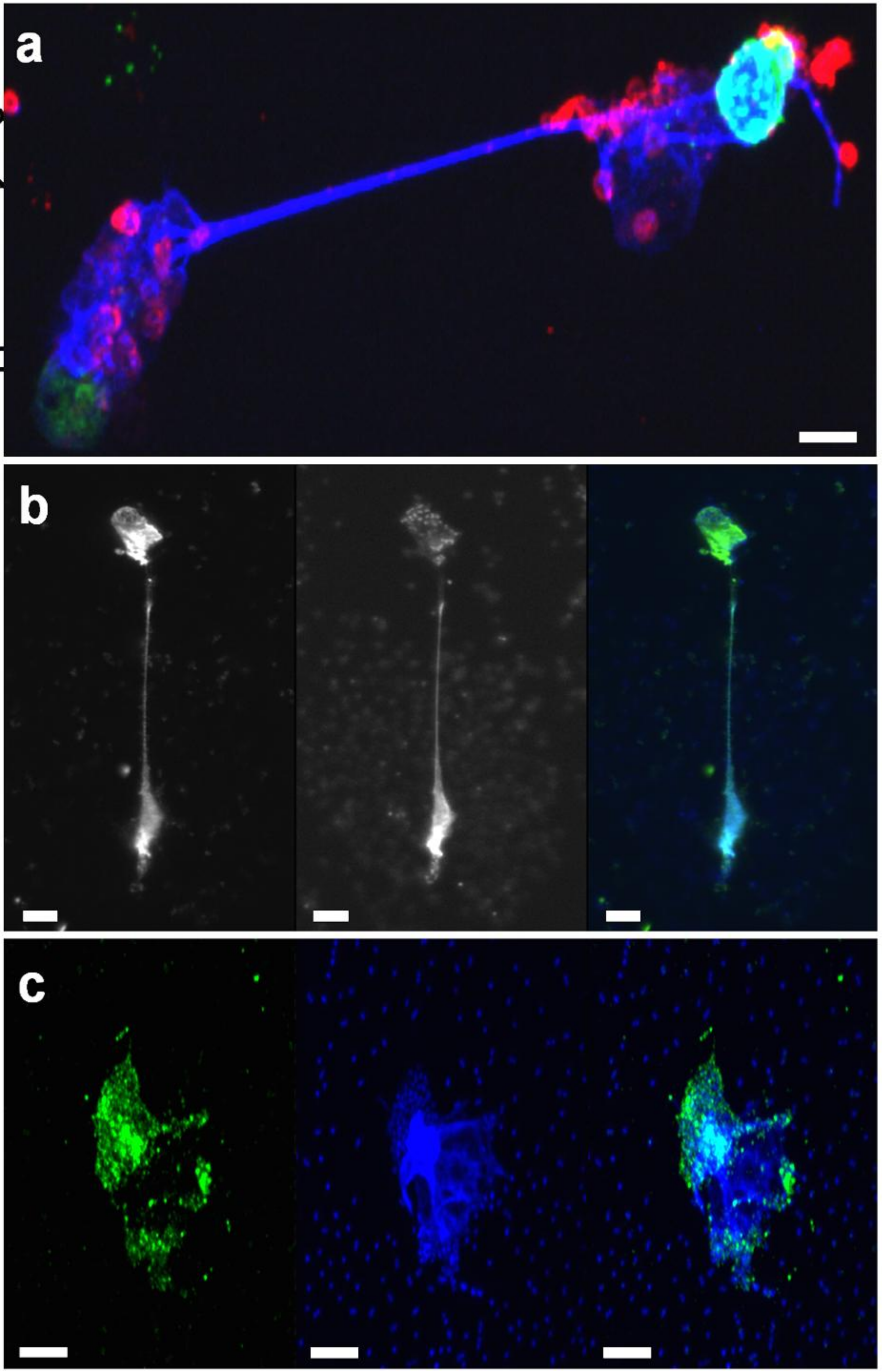

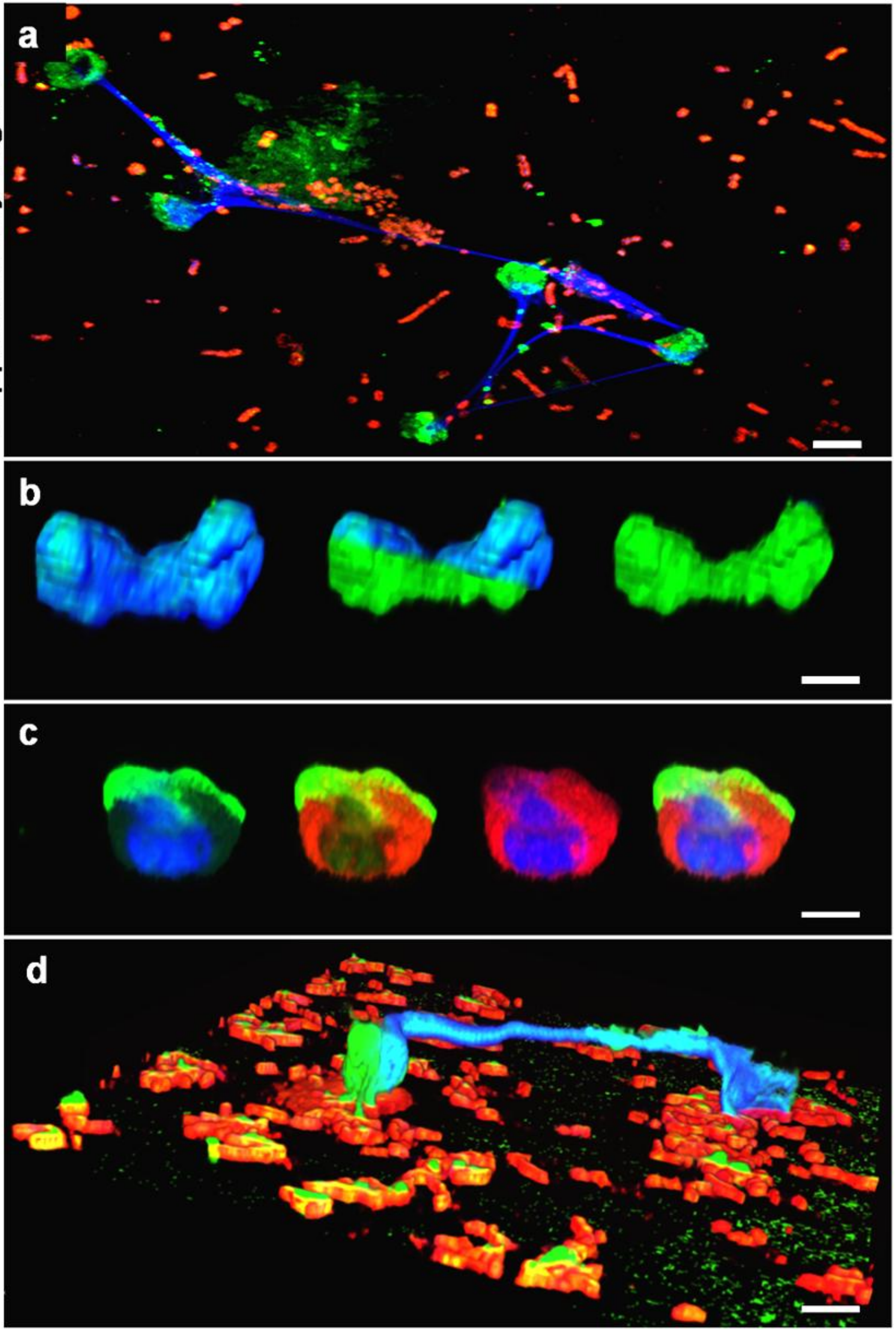

Supplementary Figure 3

Supplementary Figure 4

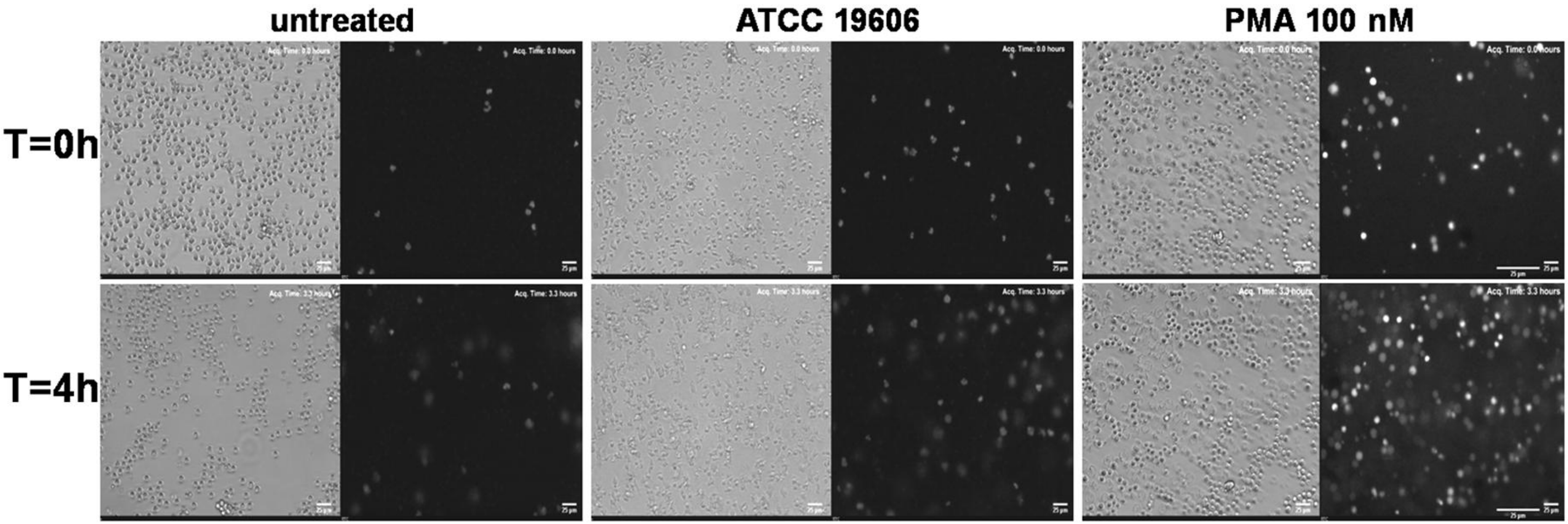

Supplementary Figure 5

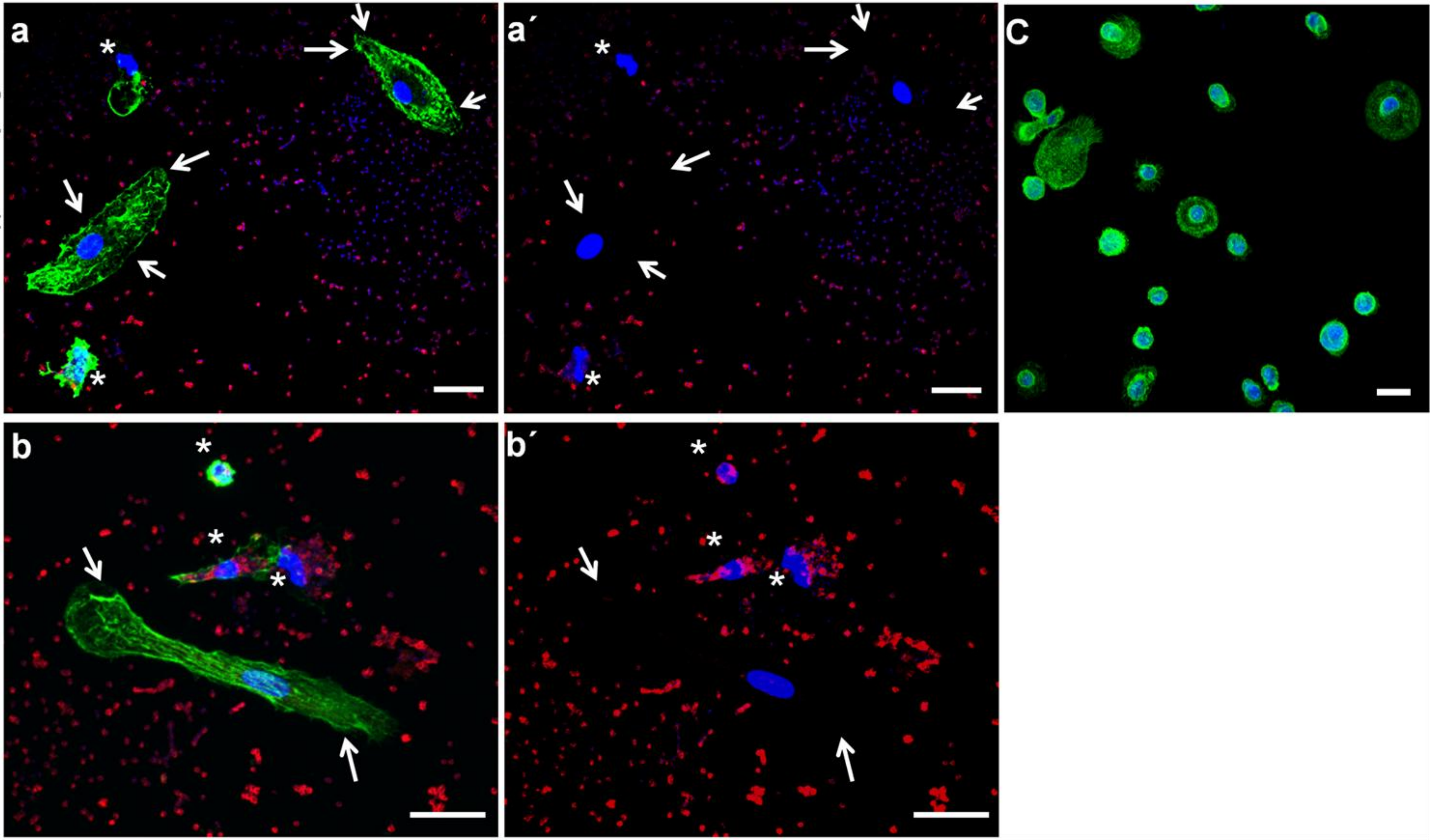

Supplement: Supplementary file 1 — Supplementary Info [file 41598_2017_4870_MOESM1_ESM.pdf]
